# Seasonal variations in the composition and diversity of gut microbiota in white-lipped deer (*Cervus albirostris*)

**DOI:** 10.7717/peerj.13753

**Published:** 2022-07-18

**Authors:** Zhangqiang You, Jing Deng, Jialin Liu, Junhua Fu, Huan Xiong, Wei Luo, Jianli Xiong

**Affiliations:** Ecological Security and Protection Key Laboratory of Sichuan Province, Mianyang Normal University, Mianyang, Sichuan Province, China

**Keywords:** 16S rRNA sequencing, Fecal, White-lipped deer, Gut microbiota, Seasonal variation

## Abstract

The gut microbiota has key physiological functions in host adaptation, although little is known about the seasonal changes in the composition and diversity of the gut microbiota in deer. In this study, seasonal variations (grassy and withering season) in the gut microbiota of white-lipped deer (*Cervus albirostris*), which lives in alpine environments, were explored through 16S rRNA high-throughput sequencing based on sixteen fecal samples collected from Gansu Qilian Mountain National Nature Reserve in China. At the phylum level, Firmicutes, Bacteroidota, and Actinobacteriota dominated the grassy season, while Firmicutes, Proteobacteria, and Actinobacteriota dominated the withering season. At the genus level, *Carnobacterium* dominated the grassy season, while *Arthrobacter* and *Acinetobacter* dominated the withering season. Alpha diversity results (Shannon: *P* = 0.01, ACE: *P* = 0.00, Chao1: *P* = 0.00) indicated that there was a difference in the diversity and richness of the gut microbiota between the two seasons, with higher diversity in the grassy season than in the withering season. Beta diversity results further indicated that there was a significant difference in the community structure between the two seasons (*P* = 0.001). In summary, the composition, diversity, and community structure of the gut microbiota showed significant seasonal variations, which could be explained by variations in the seasonal food availability, composition, diversity, and nutrition due to phenological alternations. The results of this study indicate that the gut microbiota can adapt to changes in the environment and provide the scientific basis for health assessment of white-lipped deer.

## Introduction

The gut microbiota, which refers to all microorganisms present in the gastrointestinal tract (Kim et al., 2020), has important roles in the digestion (Rowland et al., 2018), metabolism (Nicholson et al., 2012), immunity (Round & Mazmanian, 2009; Thaiss et al., 2016), and entro-endocrine function (Rastelli, Cani & Knauf, 2019) of the host. However, in a state of dysbiosis, the gut microbiota can cause many diseases, such as diarrhea (Wang et al., 2018; Xi et al., 2021), follicular cysts (Feng et al., 2021), and bacterial pneumonia (Zhao et al., 2021). Thus, the gut microbiota can affect the health of the host either positively or negatively. The composition and diversity of the gut microbiota are affected by biotic and abiotic factors, such as genetic background, diet, season, region, habitat, and other environmental factors (*e.g.*, Xin et al., 2019; Chang et al., 2020; Fan et al., 2020; Aricha et al., 2021; Guo et al., 2021; Ilina et al., 2021; Jiang et al., 2022), but diet is considered one of the predominant factors influencing the diversity and composition of the gut microbial community (Kim et al., 2014; Khafipour et al., 2016; Guo et al., 2021; Wei et al., 2021).

In recent years, variations of the gut microbiota of herbivores have been widely studied. For example, different seasons can influence the composition and diversity of the gut microbiota of American bison (*Bison bison*) (Bergmann et al., 2015), Musk deer (*Moschus berezovskii* and *M. chrysogaster*) (Hu et al., 2018; Jiang et al., 2021), Blue sheep (*Pseudois nayaur*) (Wu, 2020), yaks (*Bos grunniens*) and Tibetan sheep (*Ovis aries*) (Wei et al., 2021). Captivity can shift the diversity of fecal bacteria of Sika deer (*Cervus Nippon hortulorum*) (Guan et al., 2017) and Père David’s deer (*Elaphurus davidianus*) (Wang et al., 2019). Different regions can also influence the composition and diversity of the gut microbiota of *E. davidianus* (Zhang et al., 2018a; Zhang et al., 2018b). Winter enclosures alter the microbial communities of red deer (*C. elaphus*) (Menke et al., 2019), while diet (crop-raiding versus noncrop-raiding) and habitat (forest versus savanna) influence the microbial communities of African savanna elephant (*Loxodonta africana*) (Budd et al., 2020). Provenance and sex can also influence the microbial communities of white-tailed deer (*Odocoileus virginianus*) (Minich et al., 2021). Thus, the variations in the composition and diversity of the gut microbiota in these herbivores can be associated with shifts in dietary nutrition.

White-lipped deer (*C. albirostris*), a Cervidae species endemic to China, are mainly distributed throughout Gansu, Qinghai, Tibet, Sichuan, and Yunnan Provinces in China, and they live in alpine meadows and alpine shrubs at elevations ranging from 3,500 to 5,100 m (Cai, 1988). As the historic over-exploitation, grazing competition with livestock and habitat degradation (Harris, 2015), white-lipped deer has been defined as an endangered species, and it is on the Red List of China’s Vertebrates (Jiang et al., 2016) and the Category I key National Protected Wild Animal Species List in China. The microbial composition of the gut (Li et al., 2017), rumen, reticulum, omasum, and abomasum (Wang, 2020) of white-lipped deer has been reported. Furthermore, captivity can significantly influence the composition of the gut microbiota of white-lipped deer (Li et al., 2022). However, seasonal changes of the gut microbiota in white-lipped deer have not been thoroughly investigated. In recent years, two seasonal fecal samples of white-lipped deer have been collected from the Gansu Qilian Mountain National Nature Reserve, which provides an opportunity to study the seasonal variations of the gut microbiota. Due to the unique climatic conditions of the Gansu Qilian Mountain National Nature Reserve, the division of the four seasons is not obvious; thus, one year was divided into the grassy season (May to October) and the withering season (November to the next April) based on the growth of vegetation. In the grassy season, white-lipped deer live in alpine meadows and feed on herbs, whereas in the withering season, they migrate to lower altitudes and live at the boundary of alpine meadows and alpine shrubs, where they not only feed on withered herbs but also graze on tree barks and shrubs (pers. obs. of Zhangqiang, You, 2018–2019). During these two seasons, the shapes of the fecal samples were significantly different, and showed significant environment and diets effects. The shape of fecal samples was granular in the withering season, similar to goat fecal samples, while it was pile-like in the grassy season, similar to cattle fecal samples. Thus, we wonder how the gut microbiota of white-lipped deer can adapt to the ever-changing environments and diets. Here, we explore the seasonal variations of the gut microbiota in white-lipped deer through 16S rRNA high-throughput sequencing based on fecal samples. We hypothesize that the composition and diversity of the gut microbiota differ significantly between the seasons. This study provides new insights into the evolutionary adaptation of the gut microbiota to changes in environments and diets, and provides a scientific basis for the health assessment of white-lipped deer.

## Materials and Methods

### Ethics statement

Fecal samples were collected after foraging to ensure that the white-lipped deer were devoid of human disturbance. Permission for the collection of fecal samples was obtained from A Cheng of Gansu Qilian Mountain National Nature Reserve.

### Sample collection and preservation

Fecal samples were collected from white-lipped deer in the Gansu Qilian Mountain National Nature Reserve (97°25′–103°46′E, 36°43′–39°36′N). Eight fecal samples were collected for each season (grassy season: October 1 to 10, 2018; withering season: May 1 to 10, 2019).

Fecal samples were collected from the daily patrol route of the nature reserve. To ensure that the samples came from different individuals, only one fecal sample was collected from the same fecal pile, and the distance of each fecal sample was greater than 2.0 m. The surface of each fecal sample was removed, and only the middle portion was used to ensure the freshness of the samples and to avoid air and soil contaminants. Fecal samples were initially stored in a cooler for no more than one day, and then stored at ∼196 °C in liquid nitrogen until further study. Samples did not consider the sex and age of the individuals because we did not witness the process of excretion.

### DNA extraction, 16S rRNA amplification, and sequencing

Genomic DNA was extracted from each sample using the Soil FastDNA^®^ SPIN Kit (MP Biomedicals, Santa Ana, CA, USA) following the protocol of the manufacturer. The quality and concentration of the DNA were quantified using the ND-1000 NanoDrop^®^ spectrophotometer (Thermo Fisher Scientific Inc., Waltham, MA, USA) and 1% agarose gels for electrophoresis. The universal primers 338F and 806R (5′-ACTCCTACGGGAGGCAGCAG-3′; 5′-GGACTACHVGGGTWTCTAAT-3′) were used to amplify the V3-V4 hypervariable region of the bacterial 16S ribosomal RNA gene in fecal samples by the polymerase chain reaction (Zhou et al., 2016). PCR amplifications were performed in a 20 µL volume of the reaction mixture containing 19 Phanta^®^ Max Buffer, 200 µM dNTPs, 0.5 U Phanta^®^ Max Super-Fidelity DNA Polymerase (Vazyme Biotech Co., Ltd, Beijing, China), 0.2 µM forward primer, 0.2 µM reverse primer, and 10 ng of the DNA template (Li et al., 2017). Thermal cycling consisted of an initial denaturation at 95 °C for 5 min, followed by 30 cycles of denaturation at 95 °C for 15 s, annealing at 50 °C for 15 s, and elongation at 72 °C for 15 s. A final extension at 72 °C for 5 min was included at the end of the thermal cycling protocol. PCR products were extracted from 1.5% agarose gels and purified using the EZNA^®^ Gel Extraction Kit (Omega Bio-Tek, Inc., USA). Purified products were sequenced on the Illumina MiSeq 2500 System (Illumina Inc., USA) according to a standard protocol, and 2 × 300 bp paired-end reads were generated. Sequencing procedures were delegated to a commercial company: Shanghai Majorbio Bio-Pharm Technology Co., Ltd (https://cloud.majorbio.com/).

### Data analysis

The raw data were deposited into GenBank (Accession number: PRJNA792628). All sequences were trimmed and denoised with Mothur Software (version1.30.2) using the following criteria: reads that were shorter than 200 bp, contained any ambiguous bases, and exhibited homopolymers that were longer than 8 bp were discarded (Schloss et al., 2009). Sequences were clustered into operational taxonomic units (OTUs) using a 97% identity threshold. Taxonomic analysis of the OTUs was performed by aligning each 16S rRNA gene sequence to the SILVA ribosomal RNA gene database (release 138, https://www.arb-silva.de/) using a confidence threshold of 80% (Quast et al., 2013).

Alpha diversity indices (Shannon, Simpson, abundance-based coverage (ACE), and Chao1) were calculated with Mothur Software, and the significant differences between two seasons were determined by the Wilcoxon rank-sum test. Beta diversity was assessed using principal coordinates analysis (PCoA), which was performed using QIIME (Quantitative Insights Into Microbial Ecology) Software (version 1.9.1) (Caporaso et al., 2010) based on weighted and unweighted unifrac distance matrices. Analysis of similarities (ANOSIM) was used to determine the significance of the difference in two seasons. The Wilcoxon rank-sum test was used to verify significant differences between two seasons at phylum and genus levels. Linear discriminant analysis effect size (LEfSe) (Segata et al., 2011) was applied to analyze the potential biomarkers with statistical differences between the two seasons. All statistical analyses were performed on the services platform of Majorbio Bio-Pharm Technology Co., Ltd. (Majorbio Bio-Pharm Technology Co., Ltd., Shanghai China: https://cloud.majorbio.com/). Values are presented as mean ± standard error, and the significance level used in all tests was *P* < 0.05.

## Results

### Gut microbiota profiles

**Figure 1:**
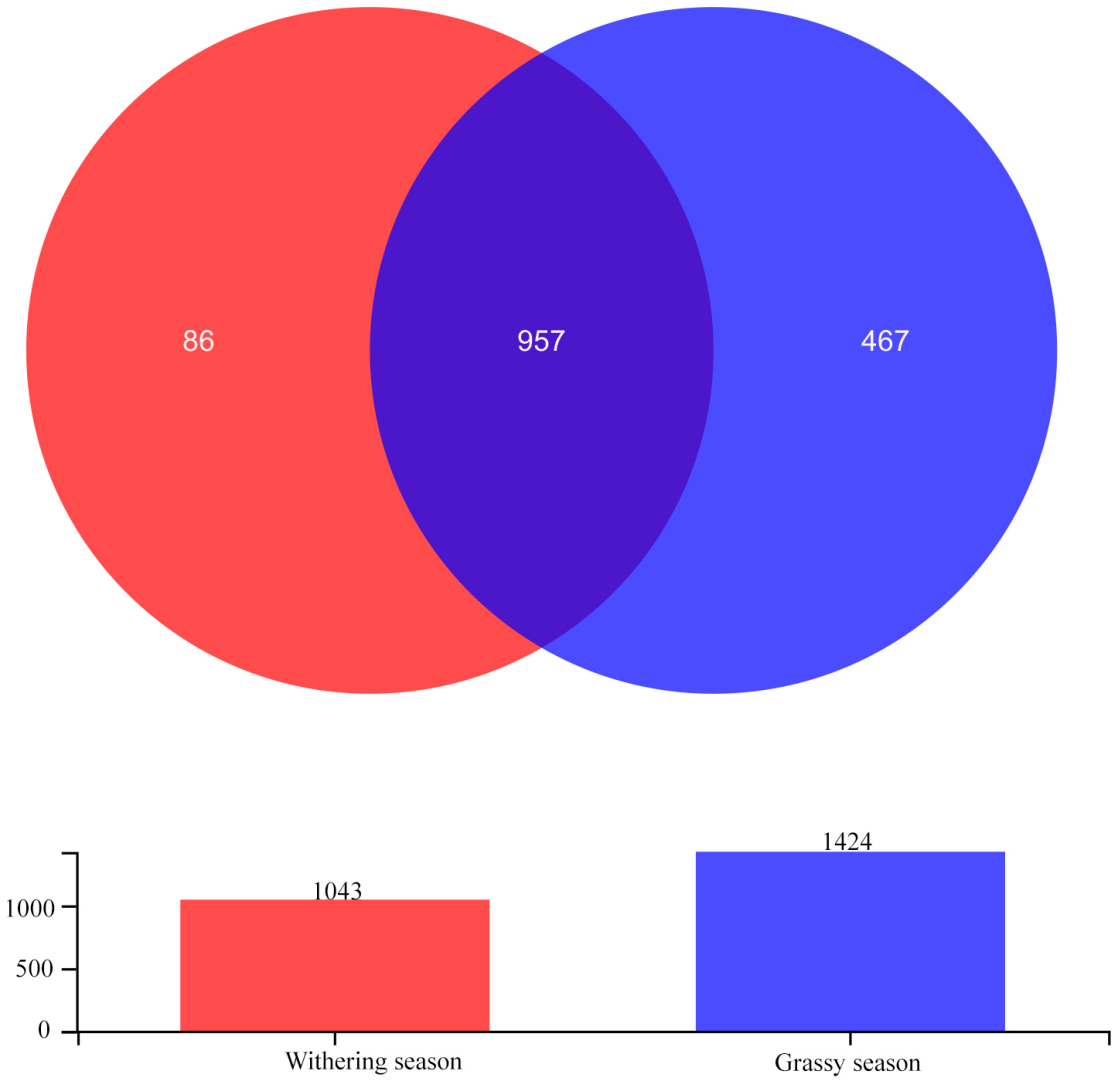
Venn diagram showing the unique and shared gut bacterial operational taxonomic units (OTUs) between grassy and withering season.

After quality filtering, a total of 906,913 high-quality sequences were obtained, with a mean of 56,682.06 sequences per sample. The sequences yielded 1,510 OTUs at a 97% similarity level with an average length of 425.78 bp per sequence. Among these OTUs, 957 OTUs were shared between seasons, and 467 and 86 were unique in the grassy and withering seasons, respectively (Fig. 1). Sobs curves, Shannon index curves, and rank abundance curves (Fig. S1) for all samples suggested that there were sufficient sequences for further analyses. The Good’s coverage (>99.78%) indicated that most gut bacterial communities of diverse species were retrieved from the samples.

### Variations in the gut microbiota diversity between grassy and withering seasons

**Figure 2:**
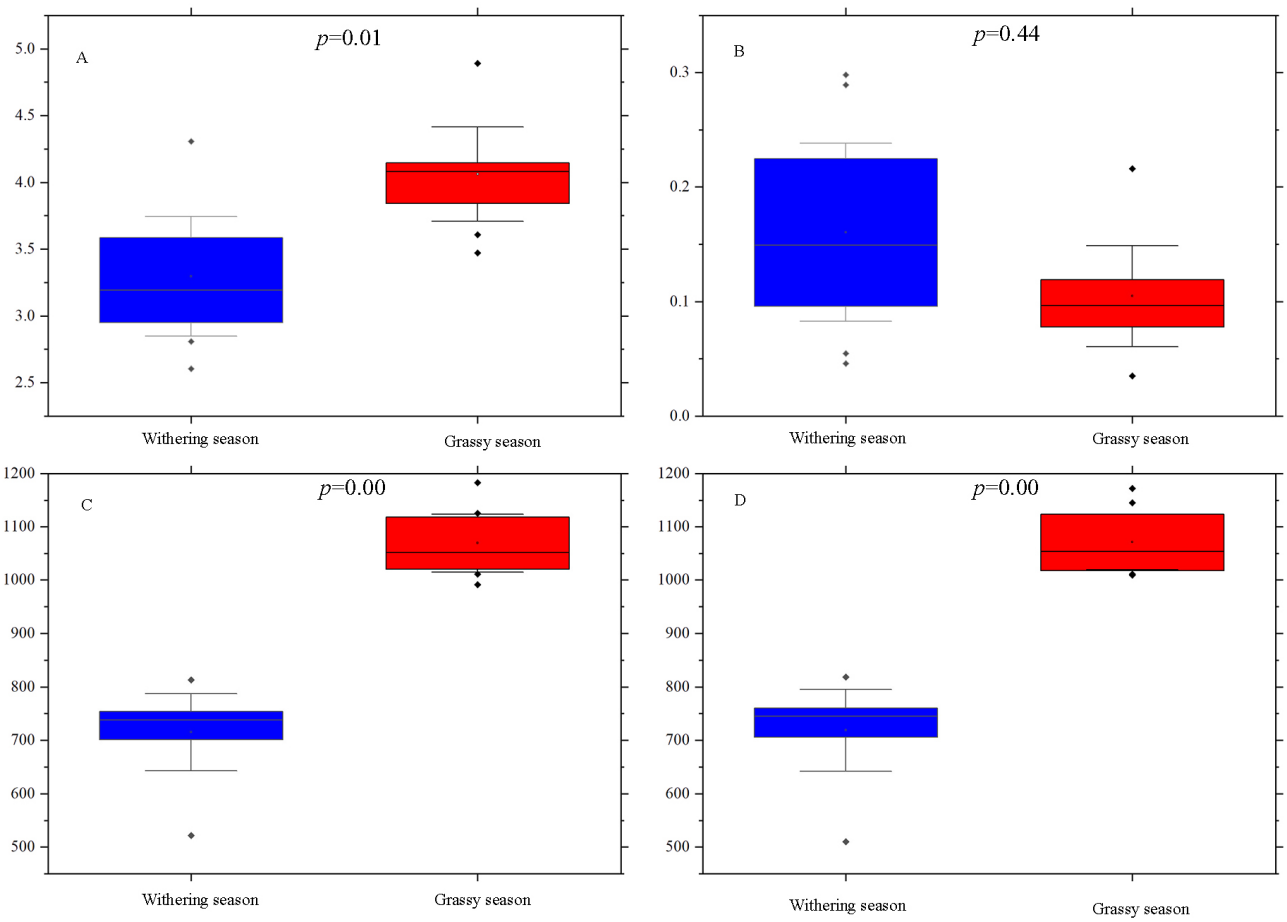
Alpha diversity indices difference analysis between grassy and withering season. (A) Shannon index, (B) Simpson index, (C) ACE, (D) Chao1. Error bars indicate standard deviation.

Alpha diversity indices, including Shannon, Simpson, ACE, and Chao1, were calculated for each season to examine whether there were diversity differences in the gut microbial community between the two seasons. The Simpson index was not significantly different (*P* = 0.44) between the two seasons (Fig. 2B), whereas Shannon (*P* = 0.01), ACE (*P* = 0.00), and Chao1 (*P* = 0.00) indices of the grassy season were significantly higher than those of the withering season (Figs. 2A, 2C, and 2D).

**Figure 3:**
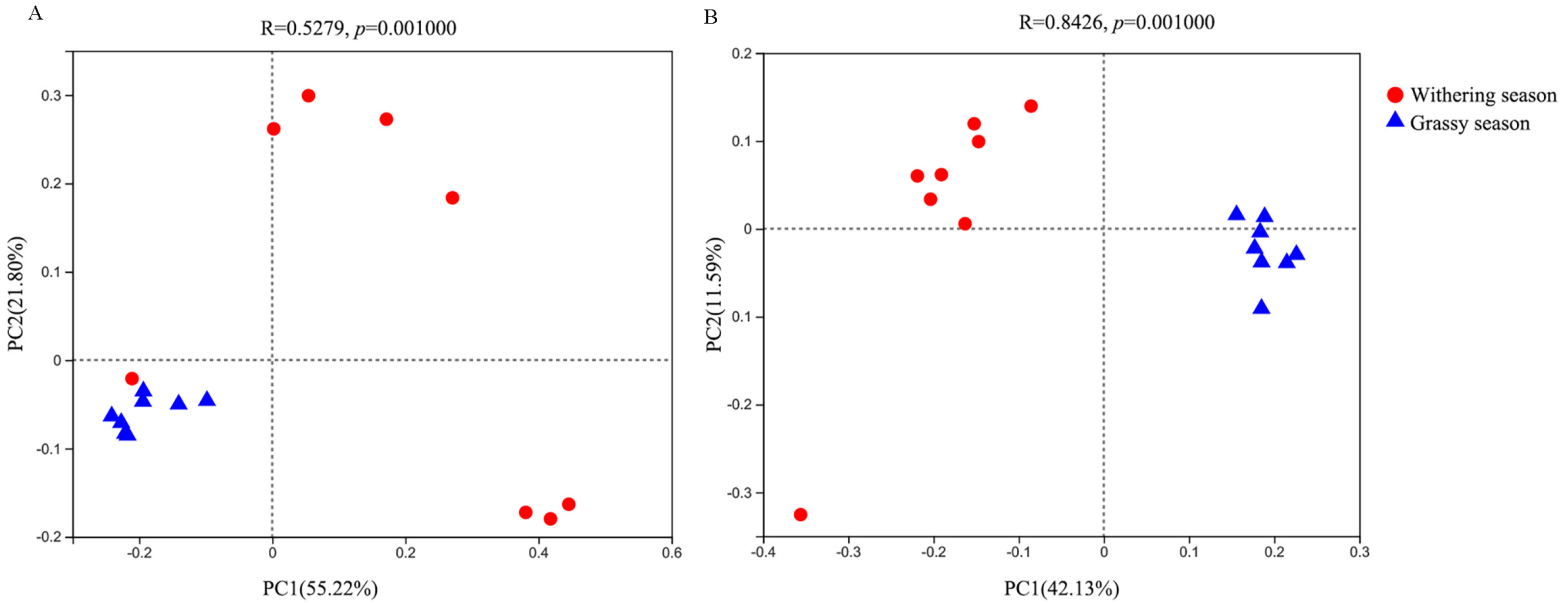
Beta diversity difference in gut microbiota between grassy and withering season. (A) Weighted-unifrac, (B) unweighted-unifrac.

With regard to the beta diversity, PCoA analysis based on weighted (Fig. 3A) and unweighted (Fig. 3B) unifrac distances was carried out to determine the differences between the two seasons. The PCoA plot showed that the samples of the grassy and withering seasons clustered separately (ANOSIM tests, weighted *R* = 0.523, *P* = 0.001; unweighted *R* = 0.843, *P* = 0.001), suggesting that there were differences in the structure of the gut microbiota in the two seasons.

### Variations in the gut microbiota composition between grassy and withering seasons

**Figure 4:**
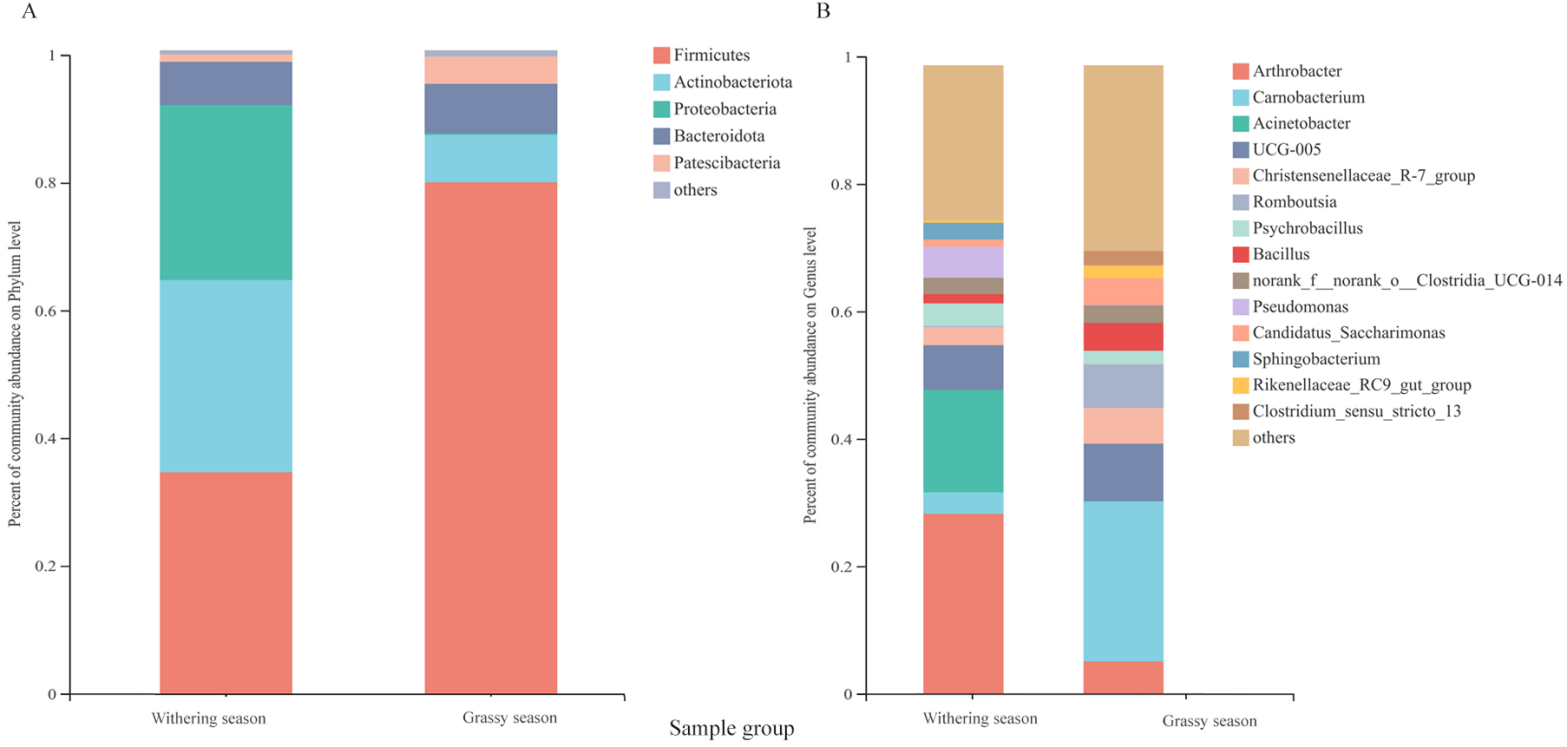
Gut microbiota composition at the phylum and genus level between grassy and withering season. (A) Phylum level, (B) genus level. Only phyla and genera with relative abundance greater than 1% are shown in the bar chart and the other taxons (<0.01%) are combined.

**Figure 5:**
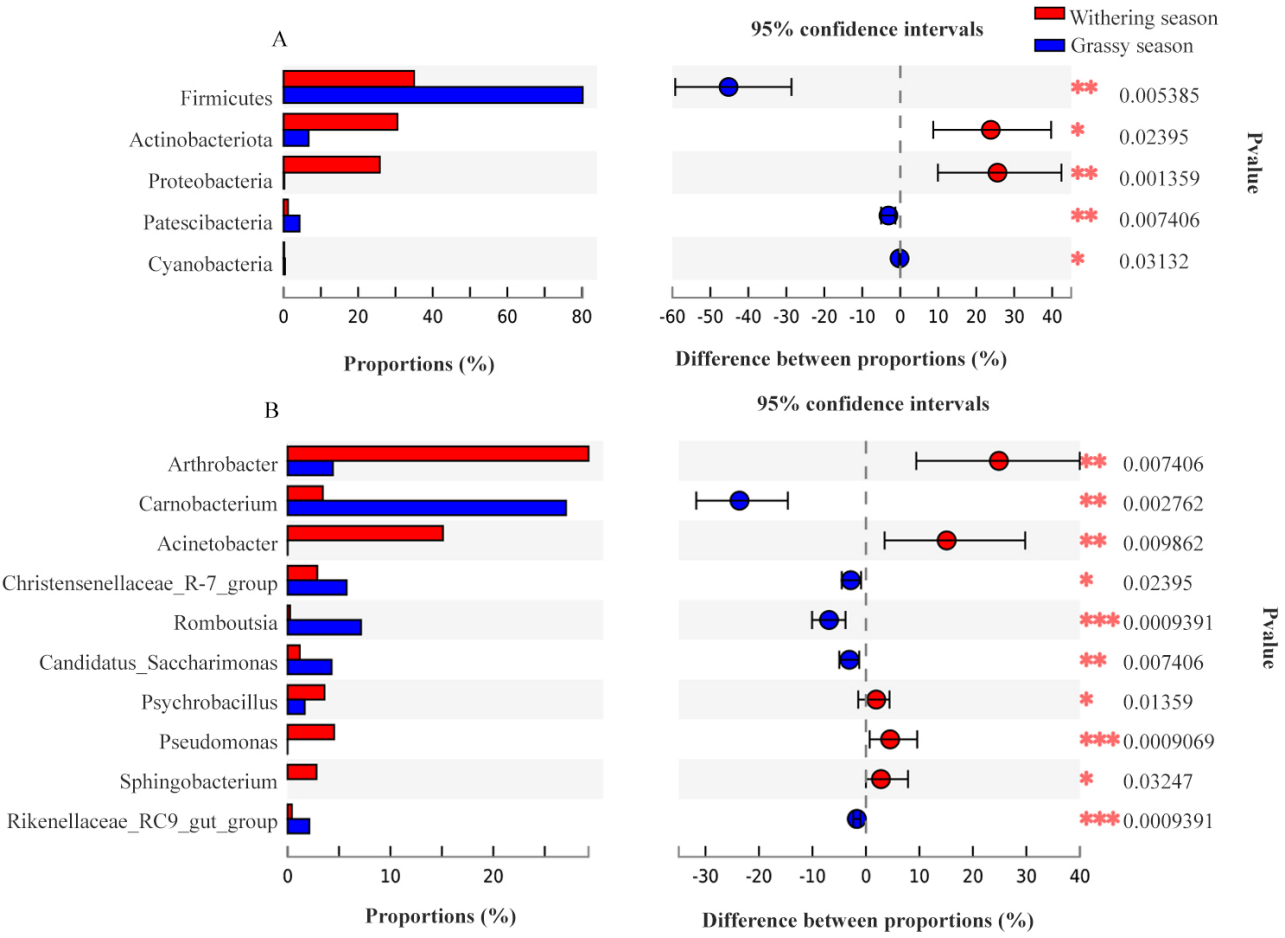
The top five phyla and ten genera with significant different between grassy and withering season. (A) Phylum level, (B) genus level.

Representatives of 574 species, 362 genera, 187 families, 106 orders, 42 classes, and 20 phyla were detected based on taxonomic assignments at a sequencing identity level of 97%. At the phylum level, the gut microbiota was mainly comprised of Firmicutes (55.18% ± 0.02%), Actinobacteria (19.52% ± 0.12%), Proteobacteria (14.78% ± 0.06%), and Bacteroidota (7.16% ± 0.00%), and these phyla accounted for approximately 96.64% of the gut microbial community (Fig. S2A). At the genus level, the gut microbiota was mainly comprised of (relative abundance, >5%) *Arthrobacter* (17.75% ± 3.94%), *Carnobacterium* (13.60% ± 0.00%), *Acinetobacter* (8.81% ± 0.79%), and Ruminococcaceae *UCG-005* (8.04% ± 0.24%) (Fig. S2B). However, the relative abundance of the predominant phyla and genera between the two seasons were different. The predominant phyla were Firmicutes (79.51% ± 0.01%), Bacteroidota (7.68% ± 0.01%), Actinobacteria (7.39% ± 0.18%), and Patescibacteria (4.25% ± 0.01%) in the grassy season, and they shifted to Firmicutes (34.41% ± 0.03%), Actinobacteria (29.87% ± 0.04%), Proteobacteria (27.22% ± 0.00%), and Bacteroidota (6.71% ± 0.00%) in the withering season (Fig. 4A). The predominant genera were *Carnobacterium* (25.49% ±0.00%) in the grassy season, and changed to *Arthrobacter* (28.57% ± 1.26%), and *Acinetobacter* (16.32% ± 0.00%) in the withering season (Fig. 4B). The top five phyla and ten genera with significant different between two seasons were compared based on the Wilcoxon rank-sum test (Fig. 5). In the grassy season, the relative abundances of Firmicutes, Patescibacteria, and Cyanobacteria were significantly higher than those in the withering season. By contrast, the relative abundances of Actinobacteriota and Proteobacteria in the grassy season were significantly lower than those in the withering season (Fig. 5A). At the genus level, the relative abundances of *Carnobacterium*, *Christensenellaccea_R-7_group*, *Romboutsia*, *Candidatus_Saccharimonas*, and *Rikenellaceae_RC9_gut_group* in the grassy season were significantly higher than those in the withering season. By contrast, the withering season had significantly higher relative abundances of *Arthrobacter*, *Acinetobacter*, *Psychrobacillus*, *Pseudomonas*, and *Sphingobacterium* compared to the grassy season (Fig. 5B).

**Figure 6:**
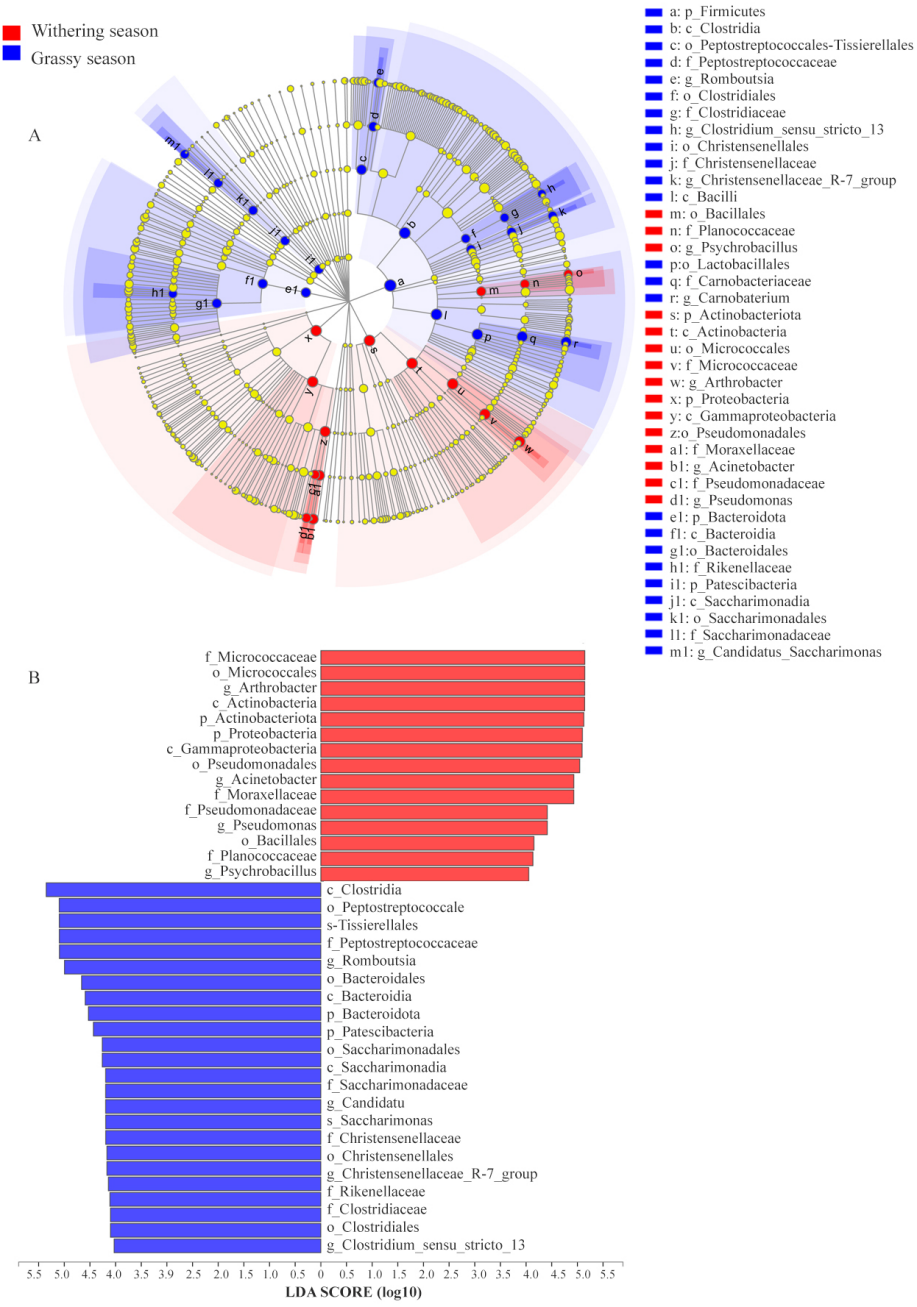
Linear discriminant analysis effect size (LEfSe) analysis of gut microbiota composition between grassy and withering season (LDA ≥ 4.0, *P* < 0.05). (A) Taxonomic representation of statistically and biologically consistent differences between grassy and withering season. Differences are represented using a colored circle, color in circles represent their respective levels of classification, and circle size is proportional to the taxon’s abundance, represents the Phylum, the class, the order, the family, and the genus. (B) Histogram of the LDA scores computed for features differentially abundant between grassy and withering season. LEfSe scores can be interpreted as the degree of consistent difference in the relative abundance of analyzed microbial communities between two seasons.

To identify the specific microbial communities that existed in each group, LEfSe analysis was conducted. Thirty-nine biomarkers were significantly different (LDA > 4.0, *p* < 0.05, Fig. 6), and fifteen and twenty-four biomarkers were presented in grassy and withering seasons, respectively. At the phyla level, Actinobacteria and Proteobacteria were significantly enriched in the withering season, whereas Firmicutes, Bacteroidota, and Patescibacteria were significantly enriched in the grassy season (Fig. 6A). At the genus level, *Arthrobacter*, *Acinetobacter*, *Pseudomonas*, and *Psychrobacillus* were significantly enriched in the withering season, whereas *Carnobacterium*, *Romboutsia*, *Candidatus_Saccharimonas*, *Christensenellaccea_R-7_group*, and *Clostridium_sensu_stricto_13* were significantly enriched in the grassy season (Fig. 6A). The core bacterial species with remarkable differences (*P* < 0.05) at all levels are shown in Fig. 6B.

## Discussion

In recent years, several studies have been published on the gut (Li et al., 2017; Li et al., 2022; Wang, 2020) microbiota of white-lipped deer, but the influence of seasons on the gut microbiota has not been investigated. This is the first study to explore the seasonal variations of the gut microbiota in white-lipped deer based on 16S rRNA gene sequencing of fecal samples. The results demonstrated that the composition and diversity of the gut microbiota, as expected, were significantly influenced by season.

Among the alpha diversity indices, Chao1 and ACE indices were used to estimate species richness, and Shannon and Simpson indices revealed species diversity (Zhang et al., 2018a; Zhang et al., 2018b). Higher Chao1 and ACE indices indicate higher richness, while a higher Shannon index and a lower Simpson index indicate higher diversity. In this study, the diversity and richness of the gut microbiota of white-lipped deer in the grassy season was higher than those of the withering season. Differences in food resources are the most direct and important factor affecting the diversity and abundance of the gut microbiota (Yun et al., 2014). Food resources can also change over temporal scales. In the grassy season, all vegetation thrives, while it withers in the withering season. For white-lipped deer, food resources and choices were plentiful in the grassy season, but relatively poor in the withering season. Furthermore, nutrient levels (*e.g.*, crude fat, crude protein, and crude fiber) in the grassy season were higher than those in the withering season (Zhang et al., 2018a; Zhang et al., 2018b; Buxton, 1996), with higher nutrient levels being more conducive to microbial fermentation (Yang et al., 2012). Although the nutritional content of the diet of white-lipped deer was not investigated, those of other species of Cervidae were examined, and the findings demonstrated that the nutritional content is influenced by different seasons. For example, the averaged winter-spring diets of *Rangifer tarandus* contained a significantly higher amount of crude fiber and lower amounts of crude protein and crude fat compared to summer-autumn diets (Yildirim et al., 2021). Thus, the diversity, richness, and high nutrient levels of food in the grassy season led to greater species richness and diversity of the gut microbiota.

At the phylum level, white-lipped deer had higher abundances of Firmicutes, Bacteroidota, and Patescibacteria in the grassy season, and higher abundances of Actinobacteria and Proteobacteria in the withering season (Figs. 4 and 5). Firmicutes can degrade various substances, which helps the host to digest and absorb certain nutrients (Kaakoush, 2015). The proportion of Firmicutes in the gut microbiota was positively correlated with the level of nutrition (De Filippo et al., 2010). Bacteroidetes is responsible for degrading carbohydrates and proteins (Fernando et al., 2010; Jami, White & Mizrahi, 2014), and it can improve the host’s nutritional outlook (Colston & Jackson, 2016). The diversity, resources, and nutrient levels of food of white-lipped deer in the grassy season were relatively rich, and the higher abundances of Firmicutes and Bacteroidetes could improve the breakdown of food (Xu et al., 2003; Backhed et al., 2005; Kaakoush, 2015), thereby generating more energy. Patescibacteria (also known as candidate phyla radiation) is ubiquitous and abundant in groundwater (Rinke et al., 2013; He et al., 2021). Drinking water is a source of Patescibacteria in the human oral cavity (Pinto et al., 2014; Bautista-de Los Santos et al., 2016). Furthermore, Patescibacteria abundance was positively correlated with ambient temperature (Qiu et al., 2020). The ambient temperature in the grassy season is higher than that of the withering season, and groundwater flows freely in the grassy season, but it freezes in the withering season. Thus, the ambient temperature and availability of groundwater for white-lipped deer may have contributed to the differences in the abundance of Patescibacteria. Furthermore, Patescibacteria abundance was positively correlated with plasma total protein, revealing that Patescibacteria may accelerate the absorption of total protein (Qiu et al., 2020). Thus, a high Patescibacteria abundance in the grassy season contributes to diets with high protein levels. Proteobacteria can promote cellulose activity, degrade a variety of aromatic compounds (Reid et al., 2011), and flexibly adjust metabolic processes to tolerate low-nutrition foods (Berg et al., 2016). The cellulolytic enzymes of Actinobacteria can promote the degradation of cellulose (Berlemont & Martiny, 2013). In the withering season, most vegetables are withered, indicating that food diversity and food resources are low. To survive, white-lipped deer must acquire food with a lower nutrient level, which has a higher proportion of crude fiber. Furthermore, white-lipped deer migrate to lower altitudes in the withering season, and the habitat shifts from alpine meadows to the boundary at alpine meadows and alpine shrubs. White-lipped deer not only feed on withered herbs but also graze on tree barks and shrubs. Thus, the food of white-lipped deer in the withering season has a higher proportion of crude fiber. A higher abundance of Proteobacteria and Actinobacteria can help to degrade crude fiber, such as lignin, in food sources (Fang et al., 2012). However, some members of the phylum Proteobacteria are common opportunistic pathogenic bacteria, which can cause a variety of diseases (Xi et al., 2021). A higher abundance of Proteobacteria in the withering season indicates that white-lipped deer are susceptible to diseases. Actinobacteria can produce many bioactive compounds (Raissa, Waturangi & Wahjuningrum, 2020), with actinomycetes producing many antibiotics that play important roles in the immunity of the host (Matsui et al., 2012). Thus, a high abundance of Actinobacteria can resist the onset of disease, while the combined actions of Proteobacteria and Actinobacteria can promote animal health. Variations in the components of the gut microbiota at the phylum level are indicative of the adaptation of the gut microbiota in relation to the changed environments and diets.

In addition, significantly different gut microbial species between two seasons were found at the genus level. For example, samples from the withering season were enriched in *Arthrobacter*, *Acinetobacter*, *Pseudomonas*, and *Psychrobacillus*, whereas those from the grassy season were enriched in *Carnobacterium*, *Romboutsia*, *Candidatus_Saccharimonas*, *Christensenellaccea_R-7_group*, and *Clostridium_sensu_stricto_13* (Figs. 5 and 6). In the grassy season temperatures were relatively high, foods were relatively rich, and nutritional levels of food were relatively high, but the opposite was true in the withering season, which lead to starvation and malnutrition of white-lipped deer in the withering season. *Arthrobacter* not only has nutritional versatility but also plays important roles in desiccation resistance, long-term starvation, and environmental stress (Niewerth et al., 2012), and enriched *Arthrobacter* can ensure white-lipped deer are not subject to these restrictions. *Acinetobacter* and *Pseudomonas*, two conditional pathogens, can cause a variety of diseases (Gauthier, 2015). In addition, some species of *Pseudomonas* play an important role in protein degradation (Liu et al., 2017). Higher abundances of *Acinetobacter* and *Pseudomonas* indicate that white-lipped deer had a higher risk of disease in the withering season, with a higher abundance of Pseudomonas compensating for the decrease of other protein-degrading microbes. *Carnobacterium* responds to thermal stress, resulting in more efficient feed assimilation (Nguyen et al., 2020). *Romboutsia* is involved in the fermentation of carbohydrates and the utilization of single amino acids (Gerritsen, 2015). *Candidatus_Saccharimonas* is associated with inflammation and the host’s immunological response (Li et al., 2021a; Li et al., 2021b). *Clostridium* can digest simple carbohydrates and complex polysaccharides (Bäckhed et al., 2004; Ramos et al., 2015; Aristilde, 2017), which may lead to increased carbohydrate metabolism (Dong et al., 2019). *Christensenellaccea_R-7_group* is involved in amino acid and lipid metabolism (Waters & Ley, 2019) and plays crucial roles in increasing blood sugar levels and promoting obesity in the host (Tavella et al., 2021). Enriched *Carnobacterium*, *Romboutsia Candidatus_Saccharimonas*, *Christensenellaccea_R-7_group*, and *Clostridium_sensu_stricto_13* can increase immunity and promote digestion in white-lipped deer.

## Conclusion

To the best of our knowledge, this is the first report on the seasonal variations of the gut microbiota in white-lipped deer. The composition and diversity of the gut microbiota in white-lipped deer showed significant seasonal variations. The seasonal variations of the composition and diversity of the gut microbiota in white-lipped deer can be explained by variations in seasonal food availability, composition, diversity, and nutrition. This study indicates that the adaptive evolution of the gut microbiota changes according to the environment and provides the scientific basis for the health assessment of white-lipped deer.

## Supplemental Information

10.7717/peerj.13753/supp-1 Supplemental Information 1 Rarefaction curves, Shannon index curves, and rank abundance curves

10.7717/peerj.13753/supp-2 Supplemental Information 2 Gut microbiota composition in all samples (A) phylum level, (B) genus level.

## Additional Information and Declarations

### Field Study Permissions

The following information was supplied relating to field study approvals (i.e., approving body and any reference numbers): Gansu Qilian Mountain National Nature Reserve approved the study. Permission for the collection of fecal samples was oral authorized by A Cheng of Gansu Qilian Mountain National Nature Reserve.

### Data Availability

The following information was supplied regarding data availability: The gut microbiota sequences are available at GenBank: PRJNA792628.
