# Supplementary figures and images for: Seasonal variations in the composition and diversity of gut microbiota in white-lipped deer (Cervus albirostris)
Source: PeerJ. 2022 Jul 18;10:e13753. doi: 10.7717/peerj.13753 (PMC9302429; doi:10.7717/peerj.13753)

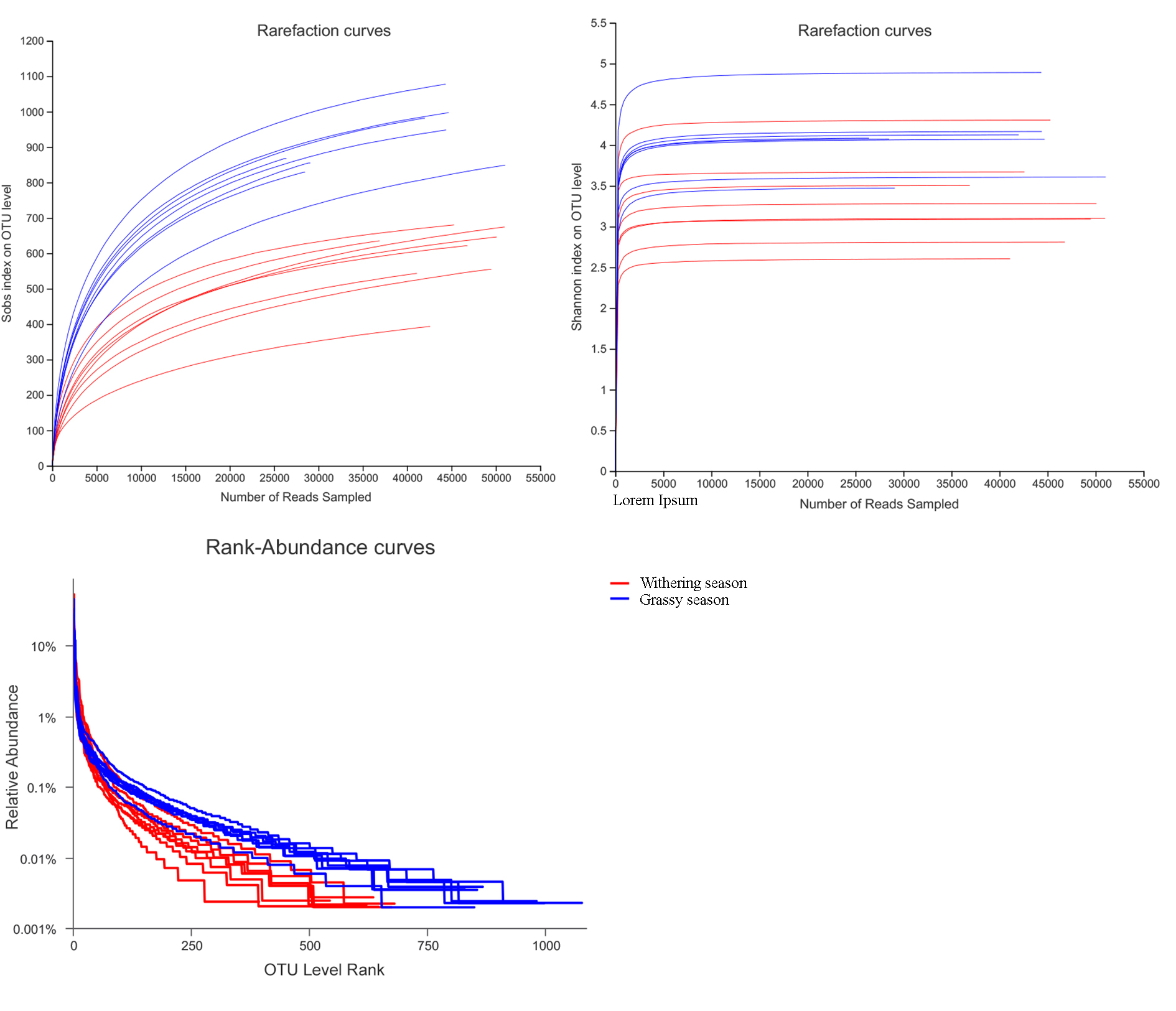

Supplement: Supplemental Information 1 [file peerj-10-13753-s001.jpg]

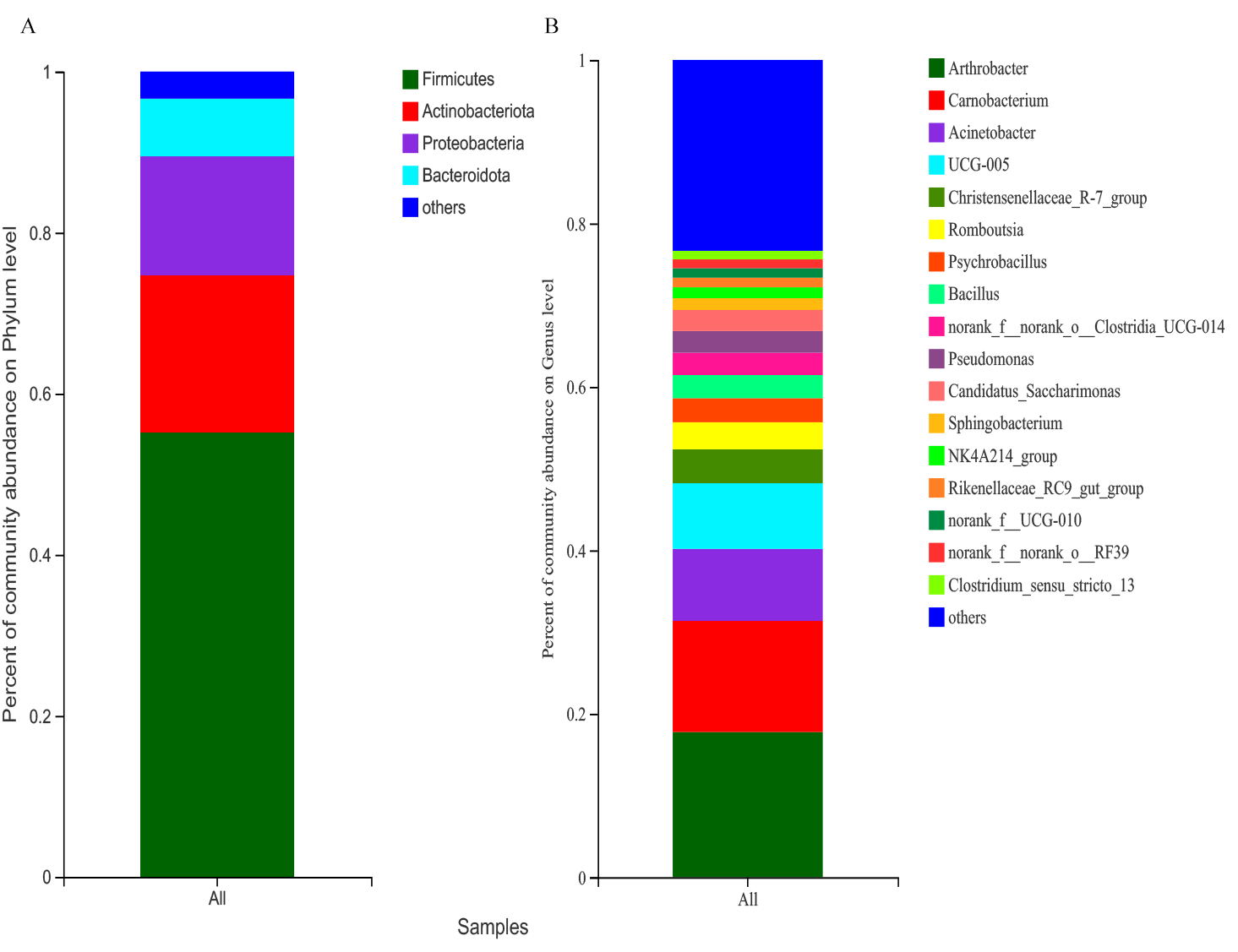

Supplement: Supplemental Information 2 — (A) phylum level, (B) genus level. [file peerj-10-13753-s002.png]
